# A high-resolution data set of fatty acid-binding protein structures. I. Dynamics of FABP4 and ligand binding

**DOI:** 10.1107/S2059798325006242

**Published:** 2025-07-28

**Authors:** Fabio Casagrande, Andreas Ehler, Dominique Burger, Joerg Benz, Alfred Ross, Markus G. Rudolph

**Affiliations:** a Therapeutic Modalities, Innovation Center Basel F. Hoffmann-La Roche Grenzacherstrasse 124 4070Basel Switzerland; University of Oxford, United Kingdom

**Keywords:** NMR dynamics, micelle and bicelle binding, residual ligands, fatty acid-binding proteins

## Abstract

Fatty acid-binding proteins (FABPs) are involved in the uptake and intracellular trafficking of fatty acids for metabolic and gene-regulatory purposes. FABPs are known to associate with membranes and also enter the nucleus. Using NMR and a human FABP4 (hFABP4) preparation completely free of endogenous ligands, we studied the influence of fatty acids and inhibitors on the conformational flexibility and bicelle/membrane association of this isoform. Binding of fatty acids and ligands rigidifies hFABP4, particularly at the portal region where ligands enter the binding site. Depending on the nature of the ligand, hFABP4 stays associated with bicelles via the portal region or segregates into solution, a prerequisite for nuclear import using a nonclassical nuclear localization signal. These results indicate that different ligands can lead to different biological outcomes. One of the major determinants for FABP4 segregation is Phe58, which in X-ray crystal structures adopts different conformations as a function of ligand volume. It is possible that other FABP isoforms use a similar mechanism for ligand-dependent membrane detachment and activation of nuclear import.

## Abstract 2

NMR studies on human FABP4 in solution and bound to micelles and bicelles delineate its dynamics in a ligand-dependent fashion. Residual ligands are co-purified with the protein, which may negatively influence binding studies with low-affinity ligands.

## 1. Introduction

Fatty acid-binding proteins (FABPs), also known as adipocyte lipid-binding proteins, are a family of ten cytosolic members with tissue-specific distribution. The term ‘lipid’ summarizes all ligands of FABPs, including, but not limited to, fatty acids. FABPs are involved in the uptake, metabolism and intra­cellular trafficking of fatty acids, thus modulating intracellular lipid homeostasis and systemic energy homeostasis (reviewed in Storch & McDermott, 2009 ▸). There seems to be little specificity for the type (saturated or unsaturated) or length (>C_14_) of fatty acid, although longer fatty acids usually display higher affinity for FABPs. Recently, FABP isoforms 3, 5 and 7 were implicated in binding to eicosanoids (arachidonic acid derivatives) and endocannabinoids (Kaczocha *et al.*, 2012 ▸), involving them in synaptic signaling (Glaser *et al.*, 2023 ▸). FABP isoforms 2, 4 and 5 may also shuttle between the cytoplasm and the nucleus. FABP5 binds specialized fatty acids and their derivatives, including retinoic acid and acyl-CoA derivatives. Upon ligand binding, a nonclassical three-dimensional nuclear localization signal (NLS) is exposed that is recognized by the importin α machinery to transport the complex from the cytosol to the nucleus (Suárez *et al.*, 2020 ▸), with the ensuing activation of nuclear receptors and gene expression (Kaczocha *et al.*, 2012 ▸; Mitchell *et al.*, 2011 ▸). Retinoic acid may also be bound to retinoic acid receptor (RAR) and retinoic X receptor (RXR), and in these complexes leads to heterodimer formation with peroxisome proliferator-activated receptor δ (PPARδ), which regulates cell proliferation, survival and apoptosis (Huang *et al.*, 2014 ▸; Bell *et al.*, 2013 ▸). Similarly, PPARγ and PPARβ activation, respectively, have been observed for fatty-acid bound FABP4 and FABP5 (Tan *et al.*, 2002 ▸; Ayers *et al.*, 2007 ▸; Armstrong *et al.*, 2014 ▸). Of the FABP isoforms, FABP4 and FABP5 have been identified as joint potential targets for the treatment of diabetes and atherosclerosis (reviewed in Furuhashi & Hotamisligil, 2008 ▸). Importantly, the heart-specific isoform FABP3 is deemed to be a counter-target as its inhibition might result in adverse cardiac effects.

**Figure 1:**
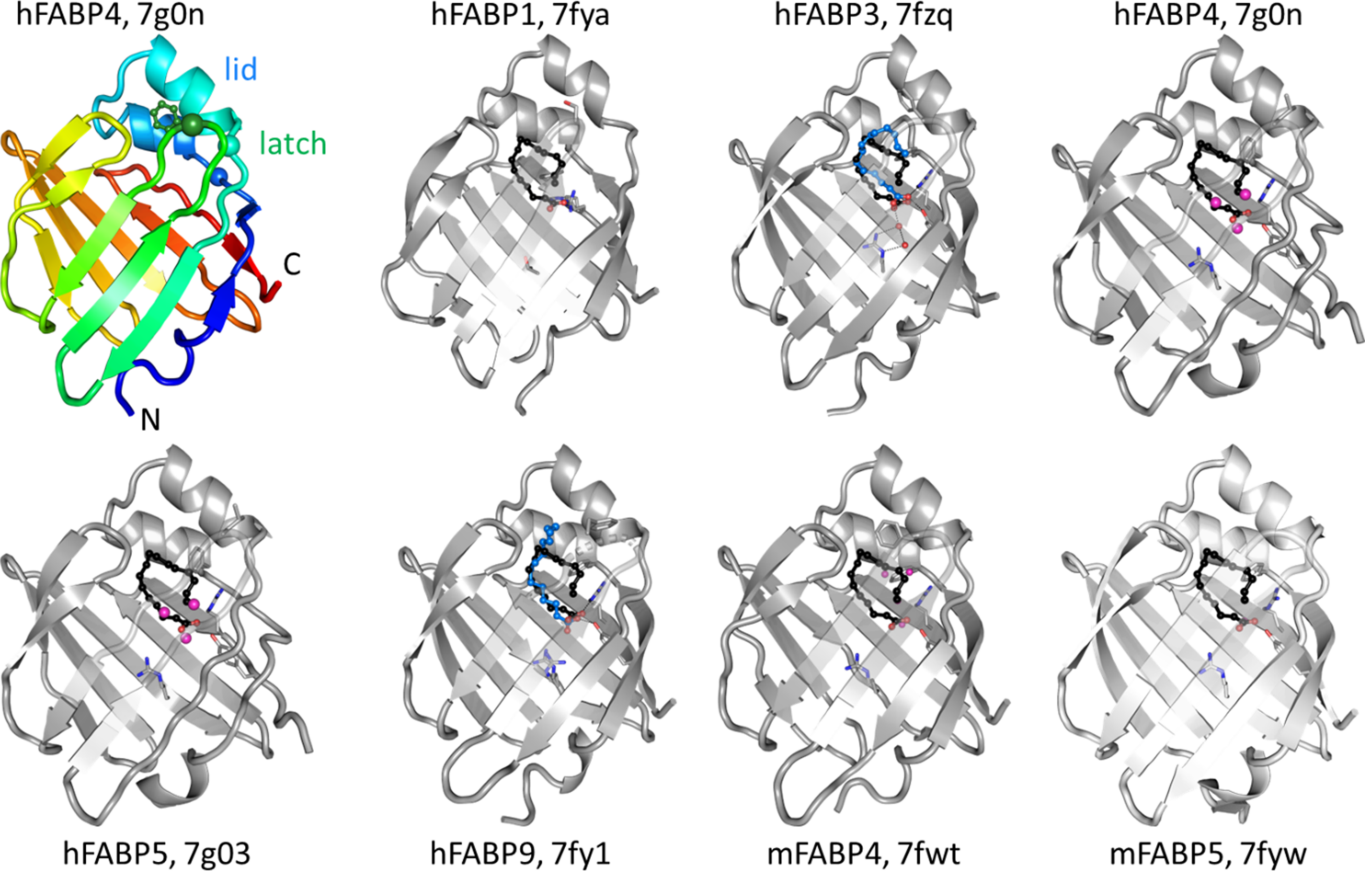
Overview of FABP isoform structures. The overall β-barrel fold of the hFABP4 structure PDB entry 7g0n is shown at the top left with the chain colored in a rainbow from the N-terminus to the C-terminus (marked). No ligand is bound in this structure, which was generated from delipidated hFABP4. The helical lid and β-hairpin latch are indicated by spheres. All structures in gray are side-by-side arrangements of the FABP isoforms discussed here in their apo or native fatty acid-bound states (blue stick model). The palmitate from PDB entry 2hnx is shown as a reference (black). Three fatty acid-interacting residues, Arg107, Arg127 and Tyr129 (hFABP4 numbering), are shown as stick models. They are conserved except for in hFABP1, which has threonine and serine at the positions of the two arginine residues.

**Figure 2:**
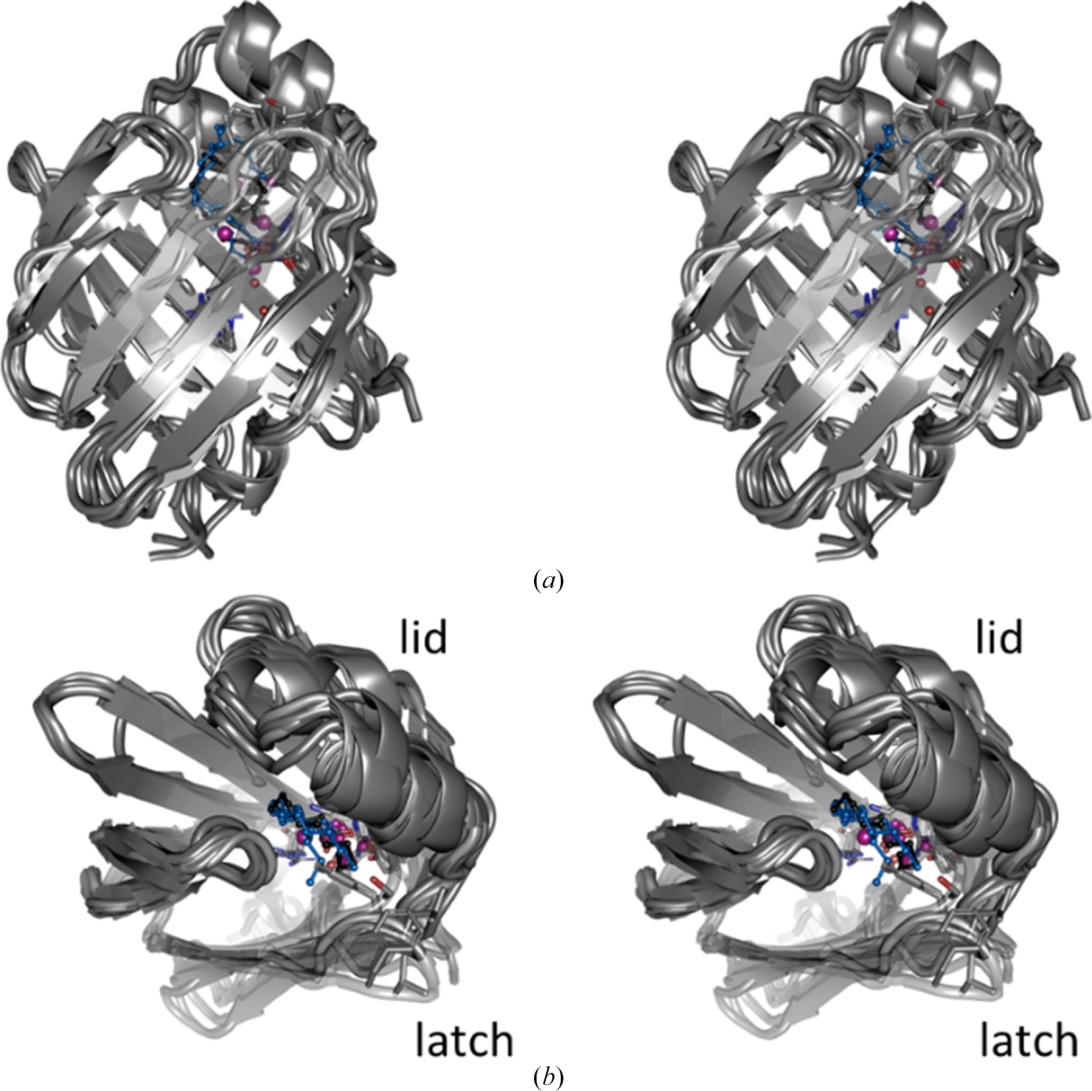
Structural plasticity in the FABP isoforms. The stereo figures in (*a*) and (*b*) are 90°-related superpositions of the isoform structures from Fig. 1 ▸. The latch residues are shown as stick modes: Ser56 in hFABP1, Phe58 in hFABP3 and hFABP4, Leu60 in hFABP5, Phe58 in hFABP9, Phe58 in mFABP4 and Val60 in mFABP5. The latch and lid have the largest structural variations within the FABP isoforms.

Together with lipocalins, FABPs belong to the calycin superfamily of proteins. While the sequence identity among FABP isoforms ranges between 20% and 70%, they share a similar overall structure, with a ten-stranded β-barrel composed of two almost orthogonal β-sheets enclosing a large interior cavity that hosts one or sometimes two long-chain fatty acids (Fig. 1 ▸). A small α-helical subdomain akin to a lid is inserted between the first and second β-strand. Opening of the lid allows entry to the barrel interior, then closes down again once the ligand has bound. The lid is locked by a residue known as the ‘latch’ to form the closed FABP–ligand complex. In FABP4, the latch is Phe58 at the end of a β-turn connecting β-strands 3 and 4 (Marr *et al.*, 2006 ▸). In other FABP isoforms, the latch is either a phenylalanine, an aliphatic residue such as leucine and valine or, exceptionally, a serine (in hFABP1; Fig. 2 ▸). Together, the lid and latch are also referred to as the ‘entrance’ or ‘portal region’ (Hotamisligil & Bernlohr, 2015 ▸).

**Figure 3:**
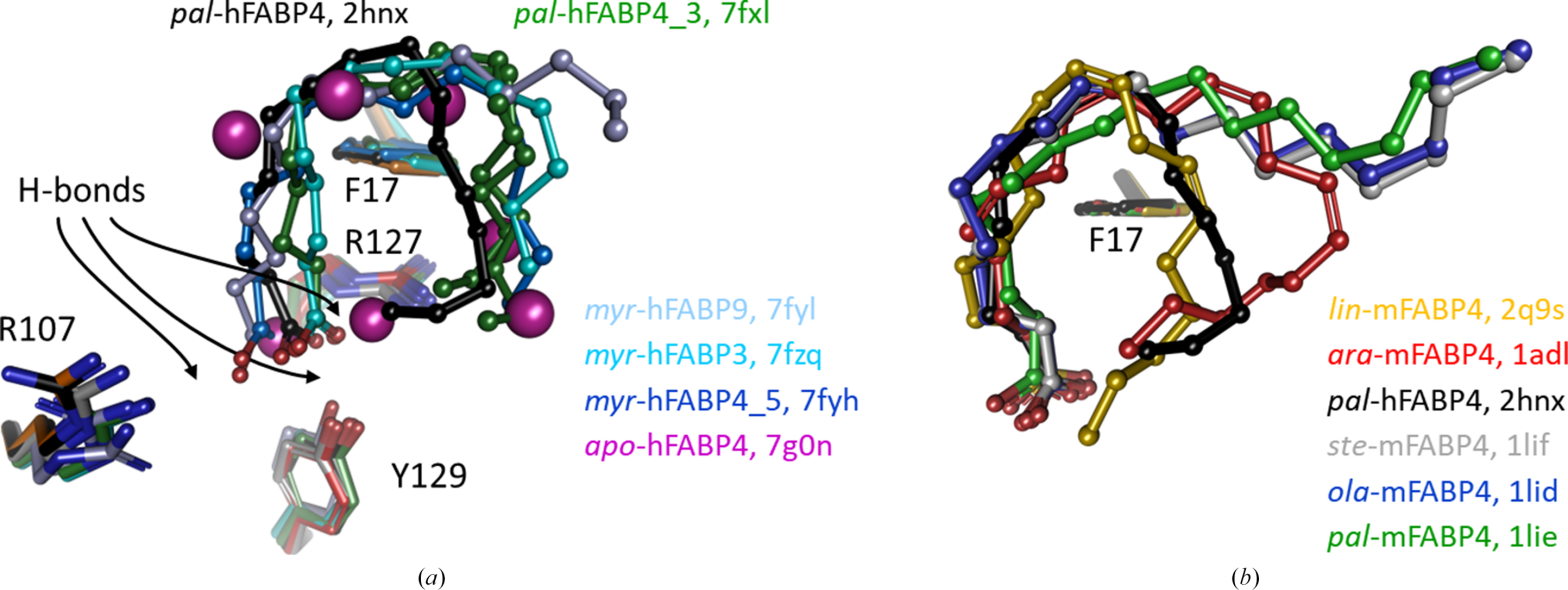
Fatty acid-binding conformations in FABPs. The orientation is the same as in Fig. 1 ▸. Fatty acids are colored according to their labels. (*a*) Comparison of different human FABP isoforms. Purified, delipidated, refolded and crystallized hFABP4 yields an apo form (magenta water molecules). Palmitate (*pal*) bound to hFABP4 (PDB entry 2hnx; black) forms a U shape as does myristate (*myr*; shades of blue) in the other isoforms, with the exception of myristate bound to hFABP9, where the fatty acid adopts the L conformation (PDB entry 7fyl; light blue). Key interacting residues are shown with numbering following that of hFABP4 and arrows point to the locations of possible hydrogen bonds between carboxylates and FABP side chains. (*b*) Comparison of U- and L-shaped conformations in human and mouse FABP4 bound to longer and unsaturated fatty acids. *pal*-hFABP4 is again shown as a reference (black). Linoleic acid (*lin*; yellow) has two *cis* double bonds and forms a tight turn. Arachidonic acid (*ara*; red) has four *cis* double bonds and adopts a wider U shape than linoleic acid. Oleic acid (*ola*; blue) with a single *cis* double bond, as well as the saturated long stearic acid (*ste*; gray), adopt the L shape, and their alkyl chains extend into bulk solvent. Of note, the shorter saturated palmitic acid adopts the L shape in mouse FABP4 (green) but an U shape in human FABP4 (black).

FABPs are co-purified in complex with their natural ligands. Crystal structures of such protein preparations revealed that the carboxylate of bound fatty acids is in contact with a conserved set of polar residues, including two arginine side chains and a tyrosine side chain, located near the bottom of the β-barrel (Fig. 3 ▸). The aliphatic tail of the fatty acid is bound by hydrophobic residues lining the inner wall of the cavity. The size and pattern of these hydrophobic residues determines the volume and shape of the cavity, allowing the development of isoform-specific inhibitors. While the carboxylate of the fatty acid is usually well resolved in electron-density maps, the aliphatic chain tends to be increasingly disordered the further away from the carboxylate the atoms are. This complicates definitive assignment of the fatty acid because the ligands in the crystal might exhibit either disorder or represent a mixture of fatty acids. In purified FABP, fatty acids consistent with myristate (14 C atoms) or palmitate (16 C atoms) are usually visible. The bound fatty acid adopts either a U-shaped or an L-shaped conformation around the tip of a conserved phenylalanine side chain (Fig. 3 ▸). The two conformations have direct consequences for the lid and latch regions of FABP. Shorter, saturated fatty acids such as myristate adopt a tight U shape that allows the lid and latch to close completely. By contrast, longer fatty acids such as palmitate can adopt either conformation. If the L shape is formed, for example in stearic or oleic acid, part of the alkyl chain contacts the lid and latch regions and extends beyond the β-barrel into bulk solvent. If longer fatty acids form the U shape, for example linoleic or arachidonic acid, the effect on the lid and latch region depends on the volume of the U conformation. Linoleic acid (18 C atoms, two *cis* double bonds) can form a tight U shape with no contact to the lid and latch region, whereas arachidonic acid (20 C atoms with four *cis* double bonds) forms a wider U shape that pushes against the lid and latch region. A consequence of a large U shape or any L shape is that the lid and latch cannot close completely. The determinants for lid and latch closure hence lie in the length and number of double bonds of the fatty acid, and this communication of bound ligand to the lid and latch, where a non­classical NLS is located (Ayers *et al.*, 2007 ▸), distinguishes the biological effect that the individual fatty acid has.

Here, we describe our crystallographic and NMR spectroscopy results on fatty-acid-bound FABP isoforms. We find that endogenous fatty acids induce conformational heterogeneity in FABP4 that can be reduced by delipidation of the protein produced in *Escherichia coli*, followed by binding of a specific fatty acid or inhibitor. We also describe a crystal structure of lipid-free human apo FABP4 at 1.12 Å resolution that harbors a water network. NMR studies using different-length fatty acids and FABP4 bound to bicelles show how FABP4 may interact with membranes to extract membrane-dissolved fatty acids for further subcellular transport. The effects of inhibitors on the flexibility and conformation of hFABP4 are also reported.

## 2. Materials and methods

Crystallographic procedures and the IC_50_ determination method are described in Ehler *et al.* (2025 ▸). Supplementary Excel File S1 lists all crystallographic and IC_50_ data values.

### 2.1. hFABP4 labeling and purification for NMR

The purification of unlabeled FABP proteins is described in the accompanying manuscript (Ehler *et al.*, 2025 ▸). For NMR studies, uniformly ^15^N and ^13^C isotopically labeled and de­lipidated FABP4 was produced. A 25 mL starter culture in standard LB medium with 100 mg L^−1^ ampicillin was inoculated with a single colony of *E. coli* BL21 (DE3) cells freshly transformed with pET-15b-His_6_-Thr-hFABP4 and shaken overnight at 37°C. This culture was mixed with 1 L M9 minimal medium (6 g L^−1^ Na_2_HPO_4_, 3 g L^−1^ KH_2_PO_4_, 0.5 g L^−1^ NaCl, 2 m*M* MgSO_4_, 1 m*M* CaCl_2_, 0.05 m*M* thiamine, 0.05 g ampicillin pH 7.0) supplemented with 1 g L^−1^^15^NH_4_Cl and 4 g L^−1^^13^C-glucose. After the culture shaken at 37°C reached an OD_600_ of 0.5 (typically after around 4 h), the temperature was lowered to 22°C and protein production was induced with a final concentration of 0.1 m*M* isopropyl β-d-1-thiogalactopyranoside. After 18 h of further incubation, the cells were harvested by centrifugation at 2000*g* and 4°C for 20 min and frozen at −80°C until use. Purification was essentially performed as described for the unlabeled proteins. Briefly, the cells were resuspended in buffer *A* (5 m*M* Tris–HCl pH 8.0, 300 m*M* NaCl) plus 5 m*M* diisopropyl fluorophosphate, 2 m*M* MgCl_2_ and a spatula tip of DNase, disrupted, centrifuged, sterile filtered and affinity chromatographed on a 1 mL HisTrap HP Ni^2+^–NTA affinity column (GE Healthcare). After washing with buffer *A* supplemented with 30 m*M* imidazole (twice the volume of loaded supernatant), His-tagged protein was eluted with 400 m*M* imidazole. Protein-containing fractions were pooled and dialyzed for 4 h against buffer *A* in a Slide-A-Lyzer dialysis cassette (3–12 mL, 3500 molecular-mass cutoff). His_6_-tags were removed by treating the dialyzed sample with 2 × 500 U thrombin plus 2.5 m*M* CaCl_2_ overnight at 4°C. Proteolysis was monitored by SDS–PAGE and cleaved protein was collected as the flowthrough of a second 1 mL HisTrap HP affinity column. The total protein concentration was determined using the calculated molar extinction coefficient of ɛ_280_ = 13 980 *M*^–1^ cm^–1^. FABP4 was delipidated by unfolding concentrated samples in 20 m*M* sodium phosphate pH 7, 150 m*M* NaCl, 5 m*M* DTT, 8 *M* urea and adding 1 mL of a washed slurry of Lipidex-1000 (a Sephadex derivative), followed by gentle shaking for 2 h at 37°C. The supernatant containing the lipid-free FABP4 was applied onto a NAP-25 column (GE Healthcare) equilibrated in five column volumes of ice-cold refolding buffer consisting of 20 m*M* sodium phosphate pH 6.0, 150 m*M* NaCl, 0.01% NaN_3_). The folding, monodispersity and purity of the delipidated FABP4 was confirmed by NMR.

### 2.2. NMR studies

Solution NMR studies used 0.1 m*M* FABP4 in 20 m*M* sodium phosphate pH 6.0, 50 m*M* NaCl in 90% H_2_O/10% ^2^H_2_O. NMR data were collected at 300 K on a Bruker 600 MHz Avance II spectrometer equipped with a 5 mm cryogenic QCI(^31^P) probe head and operated using *TopSpin* 2.1 (Bruker, Fällanden). The water signal was suppressed by a 50 Hz pre-saturation of 3 s and 1.5 s for 1D and 2D spectra, respectively, during the inter-scan relaxation delay. ^15^N–^1^H HSQC spectra were acquired with sweep widths of 16 p.p.m. and 36 p.p.m. centered at 118 p.p.m. and 4.7 p.p.m. in the indirect ^15^N and direct ^1^H dimensions and 512 transients. For the backbone resonance assignment, HNCO, HN(CA)CO, HNCA, and HN(CO)CA triple-resonance experiments (Ikura *et al.*, 1990 ▸; Kay *et al.*, 2011 ▸) were performed on ^13^C/^15^N double-labeled FABP4. Typically, 2048 complex data points were collected in the direct dimension and 64 complex data points were collected in each indirect dimension. Spectral widths were typically 5400 Hz in the direct dimension, 1600 Hz in the nitrogen dimension, 1100 Hz in the carbonyl dimension and 3400 Hz for the C^α^ dimension. Data were processed using *TopSpin* 2.1 and analyzed using *NMRview* (Johnson & Blevins, 1994 ▸). Following the analysis of delipidated (apo) hFABP4, separate experiments were conducted by adding 0.5 m*M* unlabeled fatty acid (oleic acid, arachidonic acid, and linoleic acid) and small-molecule inhibitors. The chemical shift perturbations, Δδ, induced by binding of these molecules to hFABP4 were calculated using the equation
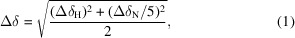
where Δδ_H_ is the change in the backbone amide proton chemical shift and Δδ_N_ is the change in the backbone amide nitrogen chemical shift.

FABP4-containing isotropic bicelles were prepared by adding delipidated hFABP4 directly to premixed phospholipid solutions containing DMPC/DMPG and DHPC (Avanti Polar Lipids, Alabaster, Alabama, USA) with molar ratios, or *q*-values, of the long-chain/short-chain phospholipids ranging from 0.1 to 1.0 and a total lipid concentration of 5%(*w*/*v*). Sample volumes of 550 µL were placed in 5 mm NMR tubes.

The structural flexibility of hFABP4 in the presence and absence of fatty acids was probed via measuring the ^15^N transverse relaxation rates (^15^N *R*_2_) using a standard Bruker pulse sequence (hsqct2etf3gpsi3d, *TopSpin* 2.1, Bruker Biospin). Pseudo-3D ^15^N *R*_2_ experiments were acquired with ten interleaved relaxation delays with total Carr–Purcell–Meiboom–Gill (CPMG) loop lengths of 20, 200, 40, 180, 60, 160, 80, 140, 100, and 120 ms and an inter-scan delay of 1.5 s. Each interleaved experiment was performed with 236 and 1024 complex data points, acquisition times of 54 ms and 61 ms, and sweep widths of 36 p.p.m. and 14 p.p.m. centered at 118 p.p.m. and 4.7 p.p.m. in the indirect ^15^N and direct ^1^H dimensions. In five subsequent experiments the CPMG pulse frequency (ν_cpmg_) was varied by setting the echo delay to 0.45, 0.575, 0.783, 1.2, and 2.45 ms while keeping the total CPMG time constant. The pseudo-3D spectrum was split into 2D planes, a squared cosine window function was applied to both dimensions, and baseline and phase corrections were performed in *TopSpin* 2.1. Dispersion data were fitted using the rate-analysis module of *NMRview*. For all assigned residues, the apparent transverse relaxation rate (*R*_2eff_) was plotted against the CPMG pulse frequency and *R*_ex_, the exchange contribution to the transverse relaxation rate *R*_2_ (Cavanagh *et al.*, 2018 ▸; Rule & Hitchens, 2006 ▸), was obtained from *R*_2eff_ = *R*_2o_ + *R*_ex_, where *R*_2o_ is the baseline transverse relaxation rate due to non-exchange processes. Experiments were repeated with FABP4 in the presence of oleic acid and small-molecule inhibitors.

## 3. Results and discussion

Shorter fatty acids commonly bind in a tight U shape around the tip of a conserved phenylalanine side chain (Phe17 in hFABP4), allowing the lid and latch to close. Longer, bulkier and some unsaturated fatty acids tend to bind in an L shape and extend into bulk solvent, leaving a gap between the lid and latch. The aliphatic part of the fatty acid is in van der Waals contact with the hydrophobic residues lining the inner wall of the cavity in FABPs. Importantly, the carboxylate group of the fatty acid is fixed by electrostatic and/or charged hydrogen-bonding interactions with two arginine side chains and a tyrosine side chain (Fig. 3 ▸). While these polar inter­actions appear particularly strong because they are embedded in an otherwise hydrophobic environment that is shielded from bulk water, water is a common co-binding molecule in FABP–ligand complexes. The *K*_d_ values of a BODIPY-labeled C_11_ fatty acid, which is too large to fit entirely into the FABP cavity, are in the nanomolar to low-micromolar range (see Section 2), making them suitable for displacement studies. Using an acrylodan-labeled method developed with FABP2 for measuring the concentrations of free fatty acids in plasma, similarly high affinities of fatty acids to other FABP isoforms were determined (Storch & McDermott, 2009 ▸). As outlined below, FABPs are co-purified in complex with endogenous ligands that require removal as an important first step when studying the specific effect of particular fatty acids on FABPs.

### 3.1. Flexibility and conformational heterogeneity of hFABP4

**Figure 4:**
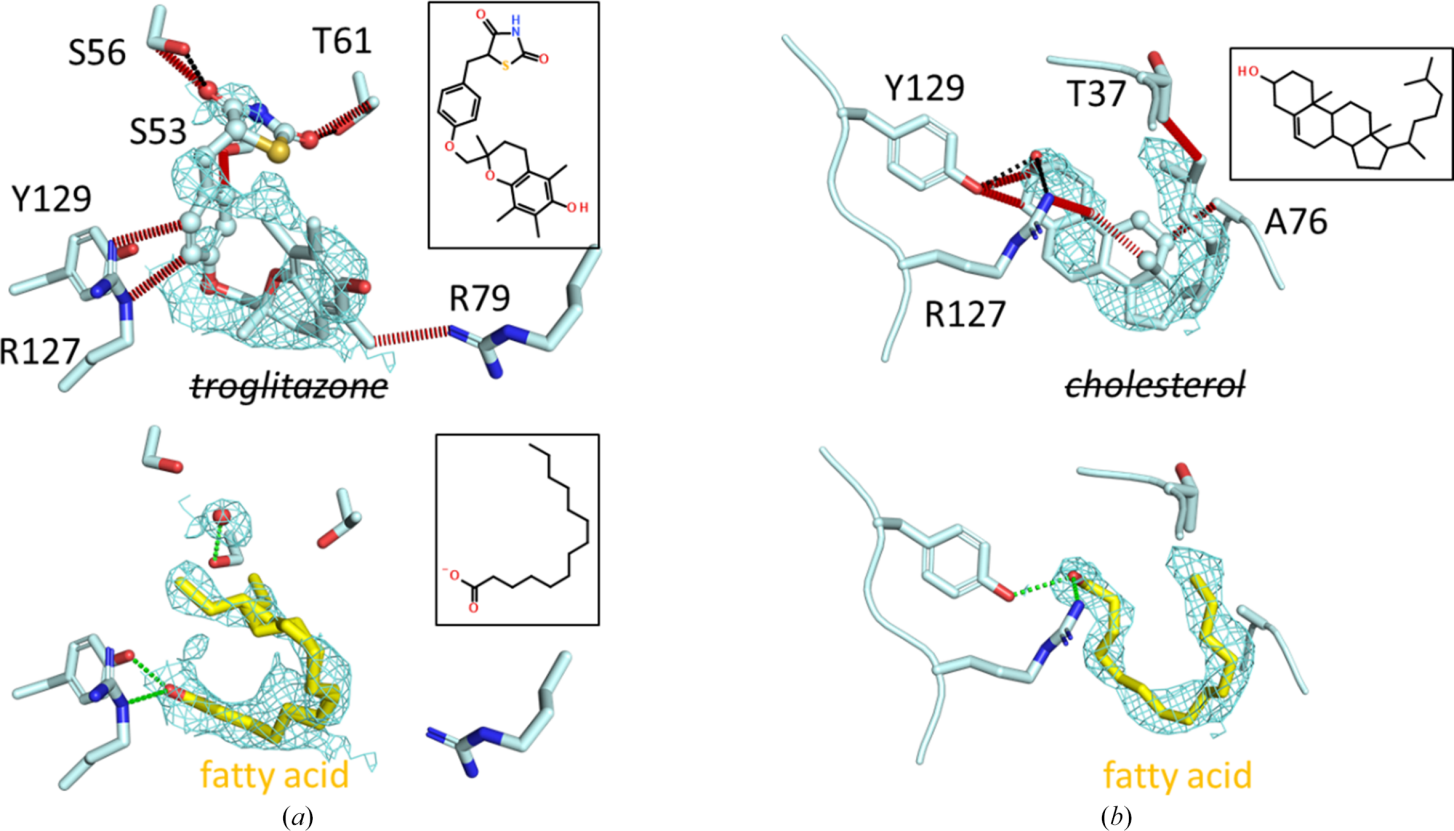
Natural ligands in FABPs that could not be replaced by other molecules. Stereochemical incompatibilities are annotated by red dashed lines. Possible hydrogen bonds are shown by black and green dashed lines, where black indicates hydrogen-bond contacts that were present in the original structure and green indicates newly formed hydrogen bonds after replacement of the ligand with a fatty acid. Atoms of the ligand that were set to zero occupancy in the original structures are shown as spheres. 2*F*_o_ − *F*_c_ electron densities are contoured at 0.8 r.m.s.d. (*a*) The hypoglycemic agent troglitazone was built into the U-shaped electron density of an endogenous fatty acid bound to mouse FABP4 (PDB entry 2qm9). About half of the ligand was set to zero occupancy. The ligand has an unusual conformation and its hydrophobic parts clash with the polar residues that normally bind the carboxylate group. Myristate (bottom) or a longer fatty acid refines well at this position, has no clashes and engages in productive hydrogen bonds (green dashes). (*b*) Cholesterol was assumed to bind to human FABP7 but is also in stereochemical conflict with the protein surroundings (PDB entry 8ivl). Three atoms within cholesterol that are located outside the observed electron density were set to zero occupancy. Again, myristate or a longer fatty acid refines well at this position. Re-refined models in complex with fatty acids and structure factors are available in the supporting information.

FABPs are usually co-purified from *E. coli* in complex with endogenous fatty acids. The ‘apo’ FABP4_5 structures with PDB codes 7fxl and 7fyh (Fig. 3 ▸) both contain fatty acids, which are modeled as myristate according to the number of C atoms that are well resolved in the electron density, although a mixture of different-length fatty acids is possible. Other FABP isoform structures, including hFABP3 in complex with modeled myristate (PDB entry 7fzq), hFABP9 in complex with modeled myristate (PDB entry 7fy1) and hFABP4_5 in complex with a fatty acid plus a small-molecule compound (PDB entries 7fyh, 7g1p, and 7g0x), support the notion that all FABP isoforms naturally contain endogenous fatty acids that must be displaced by inhibitors unless the inhibitor is so small that it can bind alongside the fatty acid. In competitive binding studies, the presence of co-purified fatty acids will shift the apparent affinity (*K*_i_ or IC_50_). Low-affinity compounds may go undetected and soaking of FABP crystals with low-affinity compounds may result in occupancies of <1 because these ligands might not be able to compete efficiently with endogenous fatty acids for the binding site. For ligand-screening purposes, such an intrinsic low-affinity filter might even be beneficial as it limits hits to those molecules that display appreciable affinity towards the fatty acid-bound FABP, which will be the predominant form *in vivo*. Two examples in which anticipated ligands have very likely been assigned to electron density from co-purified fatty acids are PDB entries 2qm9 and 8ivl (Fig. 4 ▸ and supporting information).

Protocols for removing co-purified endogenous lipids from native FABPs by hydrophobic chromatography have been developed (Wang *et al.*, 2017 ▸), but in some cases are not able to provide 100% apo forms. For NMR studies, we included a denaturation step prior to the delipidation of hFABP4 by a hydrophobic resin (Lipidex), followed by rapid folding during buffer exchange into native conditions. The unfolding/adsorption/refolding steps resulted in hFABP4 preparations that were free of detectable endogenous lipids and allowed us to delineate the effects of specific fatty acids added *in trans*on the conformational properties of hFABP4 by comparing standard and delipidated preparations of the protein.

#### 3.1.1. Residual fatty acids increase the conformational heterogeneity in hFABP4

**Figure 5:**
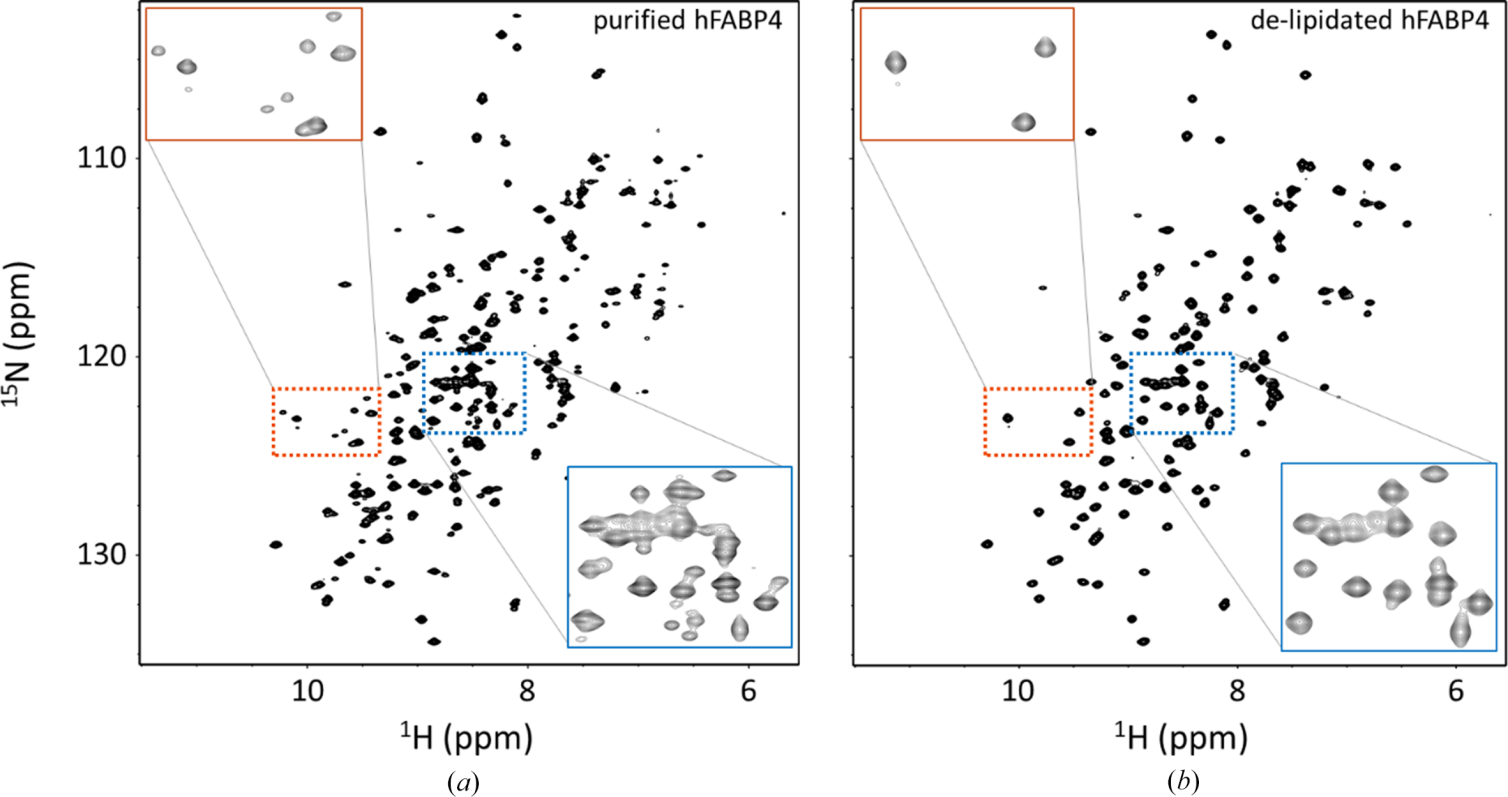
^15^N–^1^H HSQC spectra of standard purified and delipidated hFABP4. (*a*) The heterogeneity of the standard purified FABP4 is reflected by poor resolution (broader peaks) and many additional spurious peaks throughout the spectrum, indicating several states of hFABP4. (*b*) After delipidation, *i.e.* the removal of fatty acids and other hydrophobic components by denaturation, hydrophobic chromatography and refolding, hFABP4 displays a highly homogenous ^15^N–^1^H HSQC spectrum with fewer and well dispersed peaks. Blue and orange rectangles show enlargements of these spectral regions.

^15^N–^1^H HSQC NMR on standard purified hFABP4 revealed comparatively poor resolution of peaks and the presence of many additional small signals next to prominent cross-peaks (Fig. 5 ▸*a*), indicating significant conformational heterogeneity in the protein. Upon removal of endogenous fatty acids by urea-induced unfolding, hydrophobic chromatography and rapid refolding (see Section 2), the ^15^N–^1^H HSQC spectrum appeared much ‘cleaner’ and displayed fewer and much better dispersed cross-peaks (Fig. 5 ▸*b*). From these results it appears that the true apo form of hFABP4 adopts a single or very few conformations, whereas the hFABP4 preparations with a mixture of bound lipids adopt many more conformations. The dispersion and resolution of the delipidated hFABP4 ^15^N–^1^H HSQC spectrum was good enough to allow sequence assignment, to detect individual residues that change their chemical environment upon specific fatty-acid binding, and to perform relaxation analyses on micelles and bicelles, as discussed in the following.

**Figure 6:**
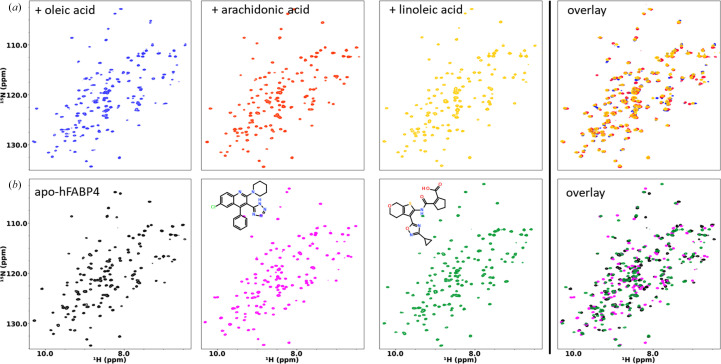
Chemical shift perturbations of hFABP4 upon fatty acid (*a*) and inhibitor (*b*) binding to apo hFABP4. The tetrazole and cyclopentenyl carboxylate (thiophene) inhibitors have IC_50_ values of 50 n*M* and 23 n*M*, respectively. Different fatty acids and inhibitors induce specific conformations in hFABP4. The right-hand panels show overlays to highlight the different conformations induced by the three fatty acids (*a*) and the two inhibitors (*b*).

**Figure 7:**
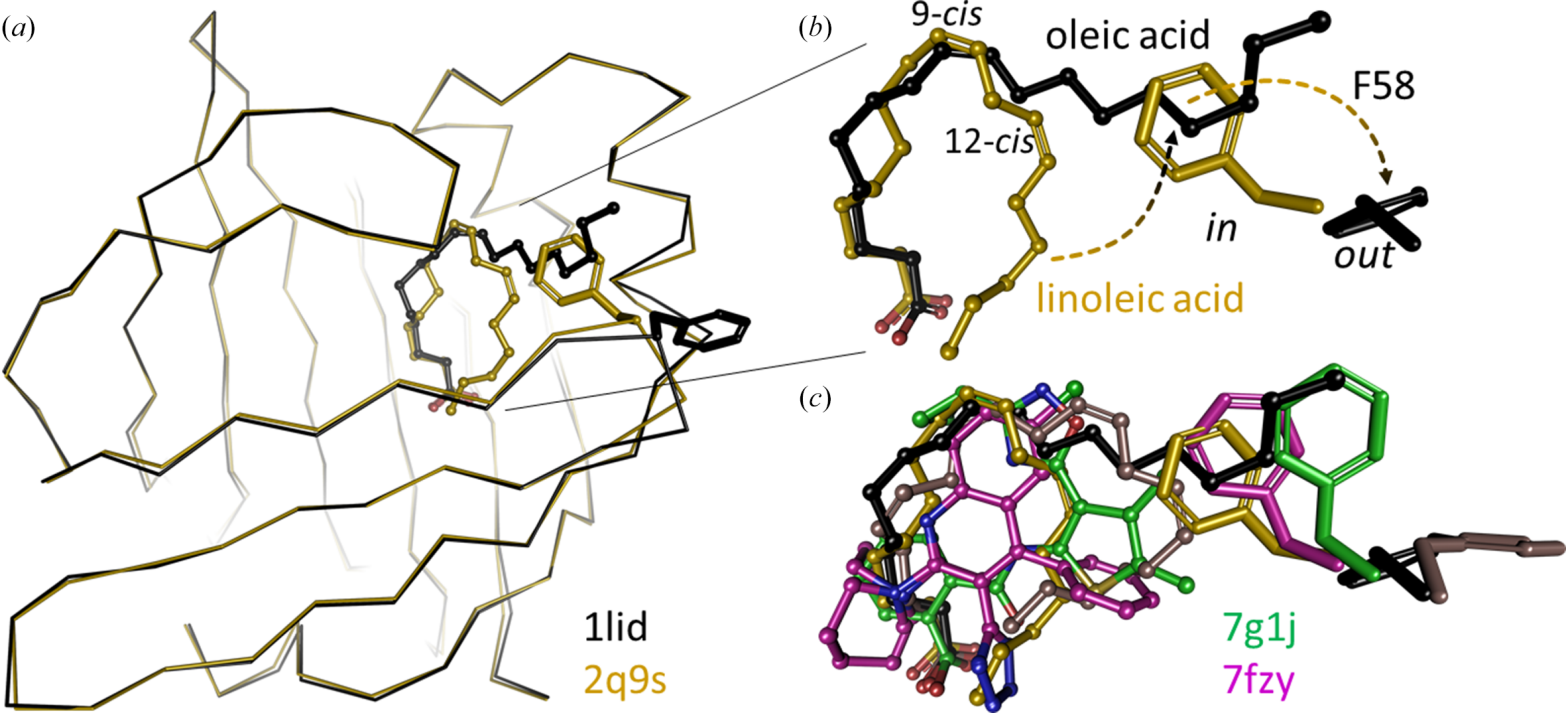
Ligand-dependent change of conformation for the latch residue Phe58 in hFABP4. (*a*, *b*) Binding of different fatty acids was shown previously to induce different conformations of Phe58. Linoleic acid (yellow, PDB entry 2q9s) has two *cis* double bonds at C atoms 9 and 12 that curve the molecule into a U shape. Oleic acid (black, PDB entry 1lid) with a single double bond at C9 reaches out into the solvent. (*b*) Enlargement of the fatty acids shows that the compact U shape of linoleic acid allows Phe58 to adopt an *in*-conformation, whereas the extended conformation of oleic acid (black) and the wider U shape of arachidonic acid [brown; shown in (*c*)] push Phe58 into the *out*-conformation. (*c*) Superposition of the ligands in the hFABP4 structures PDB entries 7g1j (green) and 7fzy (magenta) shows Phe58 to adopt intermediate conformations. Ligands in all five cases are in van der Waals contact with Phe58.

We first tested the effects that specific fatty acids have when added to delipidated hFABP4. ^15^N–^1^H HSQC spectra collected for the complexes with oleic, arachidonic, and linoleic acid show chemical shift perturbations indicative of different hFABP4 conformations (Fig. 6 ▸*a*). These fatty acids induce specific conformations, as visible from the overlay of the HSQC spectra. The crystal structures of mouse FABP4 (mFABP4) in complex with oleic, linoleic, and arachidonic acid are congruent with the notion of ligand-specific conformation (Gillilan *et al.*, 2007 ▸; Fig. 3 ▸*b*). Similarly to fatty acids, high-affinity inhibitors induce specific conformations in hFABP4 (Fig. 6 ▸*b*). Two high-affinity inhibitors of different classes, a tetrazole and a cyclopentenyl carboxylate (termed ‘thiophene’ for its characteristic middle moiety), with nanomolar IC_50_ values also induced shifts in a subset of cross-peaks in the HSQC spectra. Superposition of the crystal structures of fatty acid-bound mFABP4 and inhibitor-bound hFABP4 crystal structures show the side chain of latch residue Phe58 reacting to the nature of the bound ligand (Fig. 7 ▸). The tight U shape of linoleic acid allows Phe58 to contact linoleic acid in what may be referred to as an *in*-conformation. By contrast, the larger U shape of arachidonic acid and the L shape of oleic acid induce an *out*-conformation of Phe58. The high-affinity inhibitors induce conformations between these extremes (Fig. 7 ▸).

#### 3.1.2. Ligand binding rigidifies hFABP4, especially in the lid and latch regions

**Figure 8:**
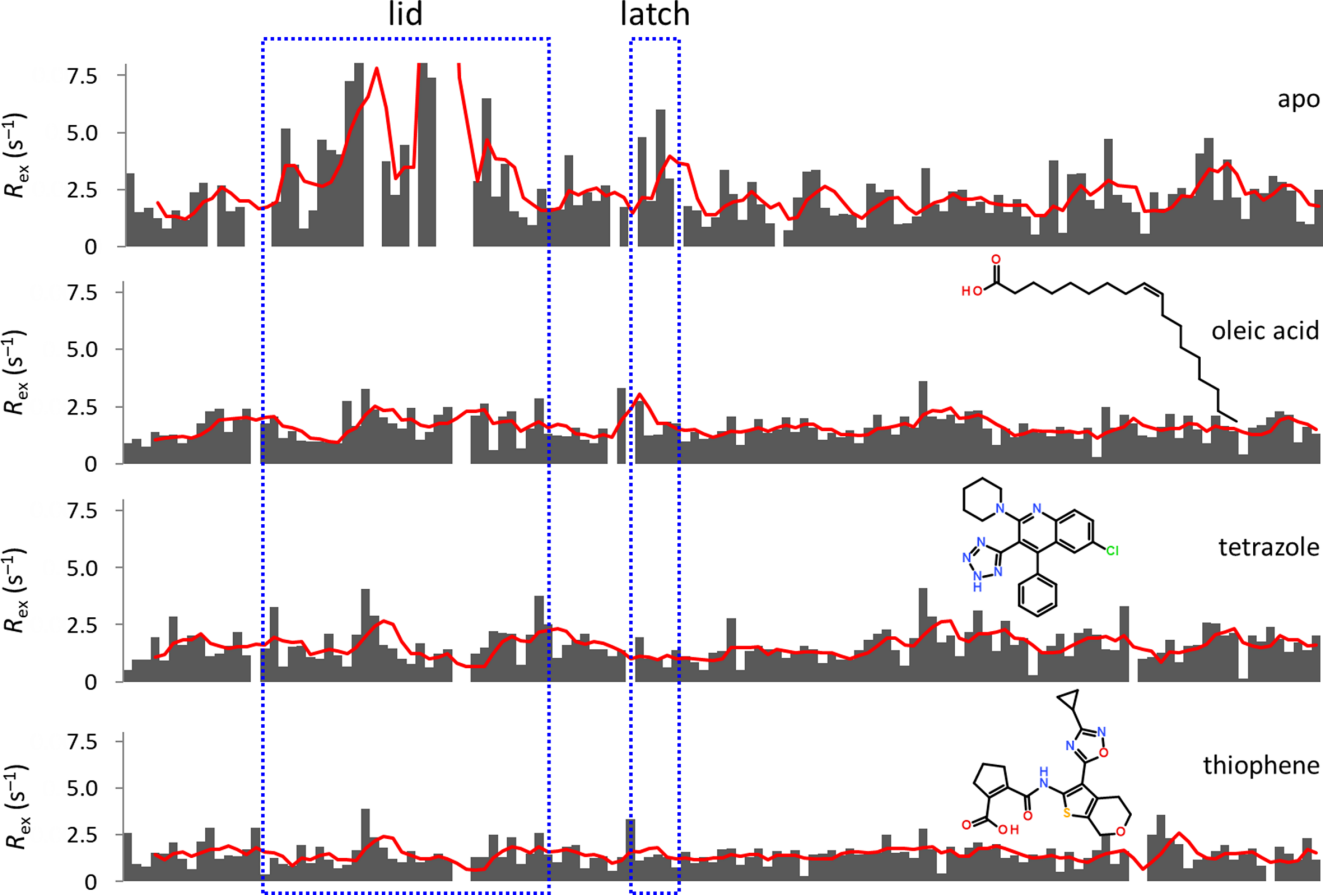
The lid and latch regions (boxed) of hFABP4 rigidify upon oleic acid and inhibitor binding. The rigidification is present along the entire sequence but is most pronounced in the lid and latch regions.

Not quite unexpectedly, binding of fatty acids and inhibitors not only induces specific conformations but reduces the flexibility of hFABP4 in general. We tested the effects of oleic acid and the two high-affinity compounds in PDB entries 7g1j and 7fzy on the flexibility of hFABP4 as expressed by the *R*_ex_ values of individual residues (Fig. 8 ▸). While the *R*_ex_ values are distributed over a wider range (0–7.5 s^–1^) in apo hFABP4, they significantly reduce to <4 s^–1^ over the entire sequence after the addition of fatty acid or inhibitors, indicating rigidification of the protein. Among these residues, the lid and latch regions show the strongest reduction in flexibility compared with the apo state. In agreement with these observations, NMR studies on FABP3 showed that different fatty acids induce ‘distinct conformational states of the protein backbone’ near the entrance or ‘portal region’ and that Phe57 in the latch region (Phe58 in hFABP4) adopts distinct conformational states depending on the chain length of the fatty acid (Lücke *et al.*, 2001 ▸). Similar results were recently obtained by NMR studies on FABP7 (Lenz *et al.*, 2023 ▸). Taken together, the crystallo­graphic and NMR data indicate that the lid and latch regions are sensitive to the ligand bound, offering a molecular mechanism to convey the information from the inside of FABP4 to its surface. However, the lid and latch regions of FABP are not completely locked after fatty-acid binding but remain flexible enough in several crystal forms to allow the soaking even of large ligands. This behavior is in line with the previous observation on FABP1 where ligand binding induced a transition of residues in the portal region from a flexible to a more ordered state with restricted motional freedom (Cai *et al.*, 2012 ▸). Since it has been suggested that FABP4 and FABP2 transiently associate with lipid bilayers via their lid region (Wootan *et al.*, 1993 ▸; Herr *et al.*, 1995 ▸; Corsico *et al.*, 1998 ▸; Liou & Storch, 2001 ▸) to facilitate ligand exchange, we first aimed to reproduce this result for hFABP4 and then to test the effect of fatty acids and inhibitors on the binding of hFABP4 to bicelles.

### 3.2. Binding of hFABP4 to bicelles as membrane surrogates

#### 3.2.1. A conserved set of residues in hFABP4 change in chemical shift upon binding to bicelles

Micelles and bicelles can be surrogates for membranes, and due to their limited size are more suited for measuring NMR relaxation times than membranes. Prepared by mixing a long-chain lipid with a short-chain lipid that functions as a mild detergent, bicelles ideally assemble into flat disks with the lipid concentrated in the center and the detergent at the rim. The molar ratio of lipid to detergent is the *q*-value which, together with the nature of the constituents and the temperature, determines the size and shape of the assembly (Caldwell *et al.*, 2018 ▸). At *q*-values of <1 the detergent concentration is higher than the lipid concentration. In the range 0.5 < *q* < 0.7 the lipid/detergent mixture assembles predominantly into bicelles with bilayer-like properties, whereas for *q* < 0.3 the aggregates are more spherical, mixed micelles without pronounced compartmentalization of their constituents (Oliver *et al.*, 2014 ▸). For solution NMR studies, bicelles with *q* < 0.7, also known as fast-tumbling or ‘isotropic’ bicelles, are preferred as they retain spectral resolution (Piai *et al.*, 2017 ▸).

**Figure 9:**
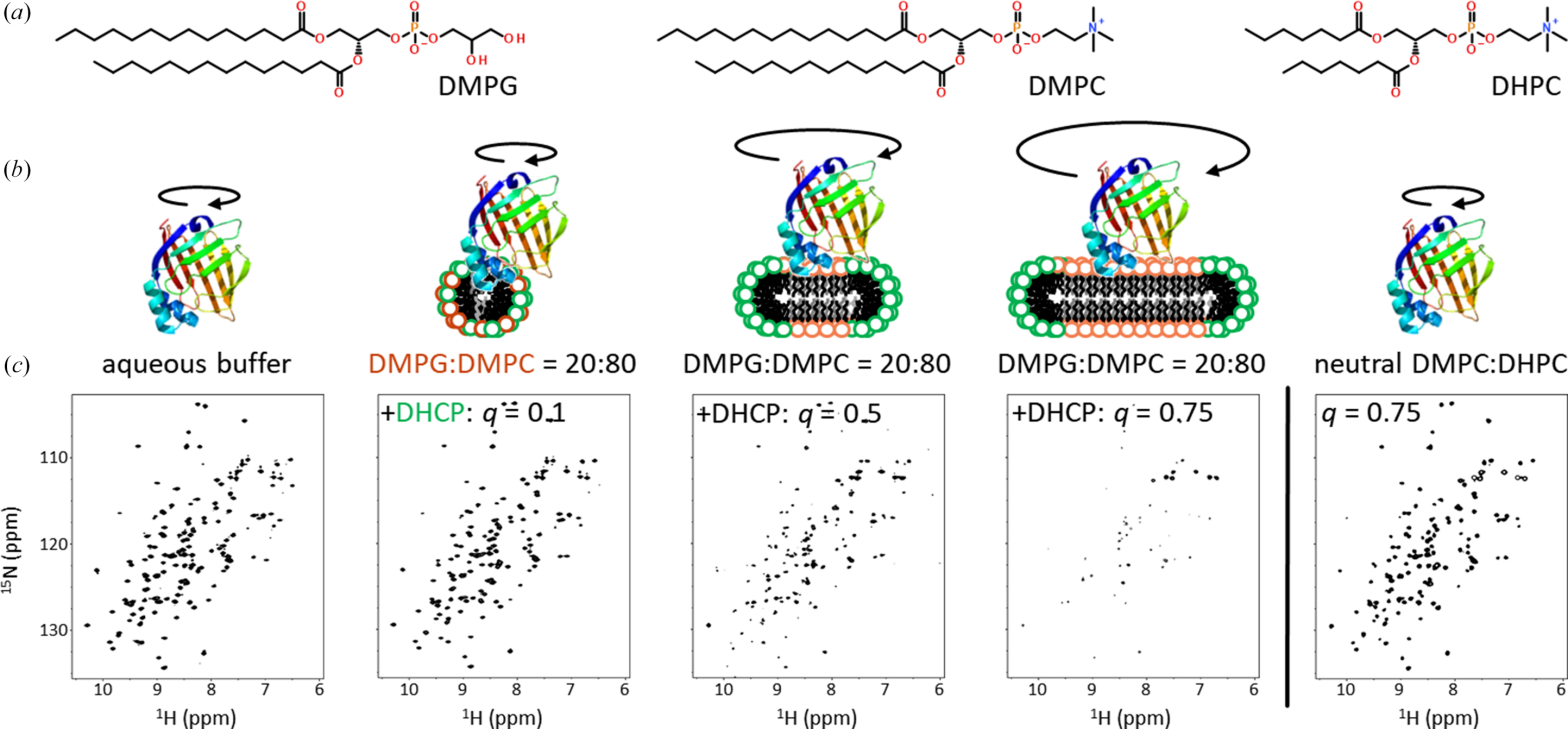
Association of hFABP4 with micelles and bicelles. (*a*) Chemical structures of the lipids and detergent used. (*b*) Schematic view of the increased hFABP4 tumbling times in the presence of micelles and bicelles. The larger the assembly, the larger the tumbling times and the smaller the peak resolution and peak heights. (*c*) A negatively charged 20:80 mixture of long-chain DMPG:DMPC lipids was titrated with the short-chain detergent DHCP to arrive at the *q*-values indicated. The HSQC spectra are contoured at the same height for comparison. The spectrum on the left is the spectrum in aqueous solution as a reference. The spectra in the middle indicate binding of hFABP4 to charged micelles (three middle spectra) with increasing bicelle character as a function of *q*. No binding is observed to neutral DMPC/DHPC bicelles (right-hand spectrum).

Bicelles were formed by mixing charged and uncharged lipids with detergent in different molar ratios *q*. The long-chain lipid DMPC and the short-chain detergent DHCP are zwitterions and hence DMPC:DHCP bicelles are uncharged overall. The lipid DMPG, on the other hand, carries a net negative charge. Charged DMPG:DMPC (20:80) bicelles with DHCP added to reach *q*-values of 0.1, 0.5, and 0.75 show distinct affinity for hFABP4 (Fig. 9 ▸). Binding of hFABP4 to bicelles will decrease the *T*_2_ relaxation times as a function of bicelle size, leading to a decrease in signal intensities and peak broadening, and thus to lower peak resolution in the ^15^N HSQC spectra. Compared with a control in the absence of any detergents or lipids, hFABP4 appears to bind to charged mixed micelles even with a small *q* of 0.1 (Fig. 9 ▸). Moreover, at *q* = 0.5 and especially at *q* = 0.75, a strong decrease in relaxation times is observed, indicating pronounced binding of hFABP4 to the charged bicelle compared with the micelle. The binding is mainly due to electrostatic attraction between hFABP4 and the bicelle, as uncharged bicelles made from DMPC:DHCP with *q* = 0.75 do not show a comparatively strong decrease in intensities but the HSQC spectra resemble those obtained in aqueous solution (Fig. 9 ▸). In summary, hFABP4 binds to charged mixed micelles and bicelles but not to neutral bicelles, pointing to a tendency for its interaction with natural membranes.

**Figure 10:**
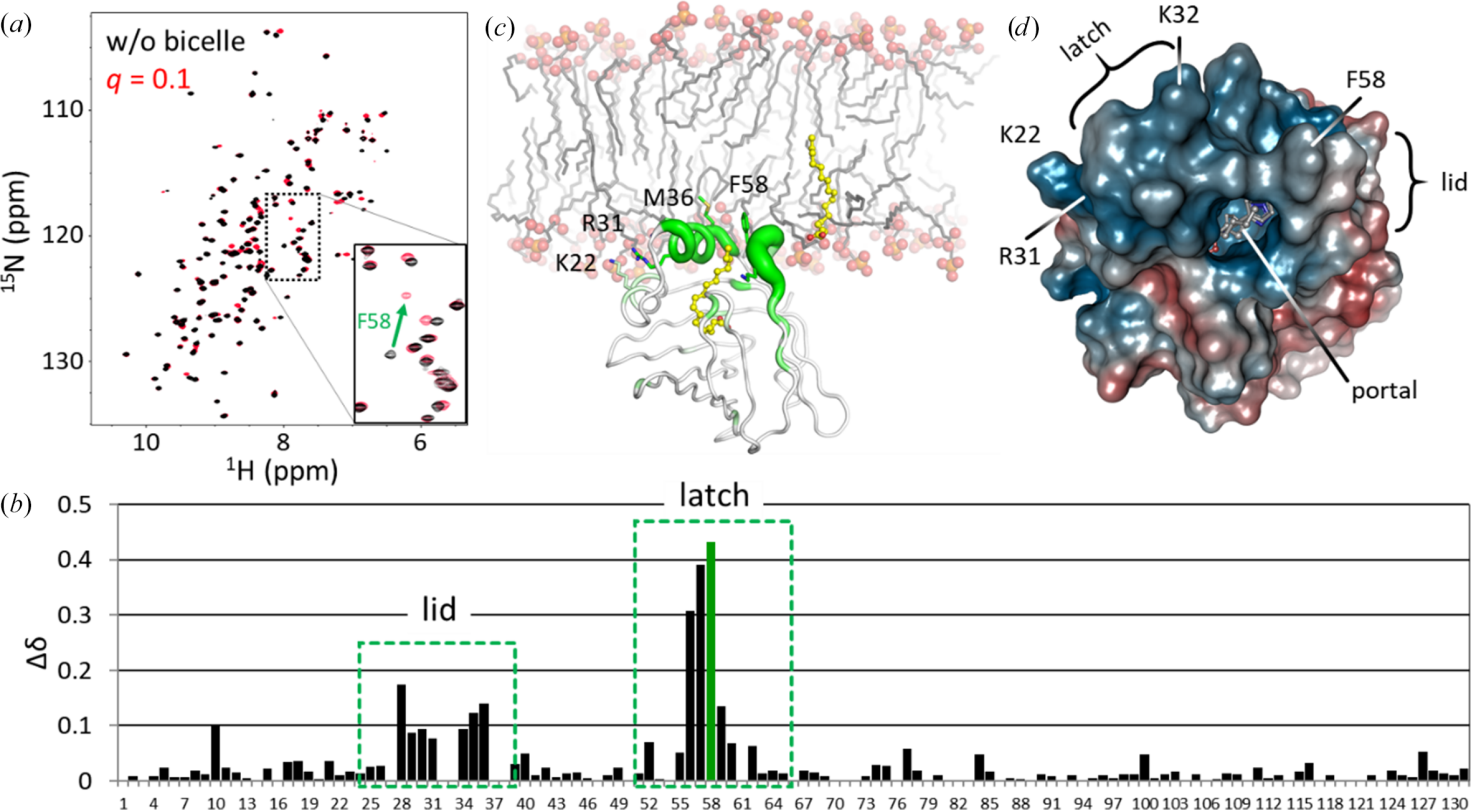
Membrane-interacting residues in hFABP4. (*a*) HSQC spectra of hFABP4 were recorded in the absence (black) and presence (red) of a DMPG:DHCP mixed micelle of *q* = 0.1. Comparison of the spectra shows chemical shifts δ even at this small *q*-value. (*b*) The plot of δ as a function of sequence position shows the lid and latch regions to exhibit the largest chemical shifts, indicating binding of these regions to the mixed micelle. (*c*) Model of FABP4 binding to a membrane (based on Bolterauer & Heller, 1996 ▸) *via* its lid and latch regions. The magnitude of the chemical shifts is shown as the thickness of the green tube. (*d*) The asymmetric charge distribution in hFABP4 supports this membrane-interaction model. The electrostatic potential of PDB entry 8s1k was calculated with *APBS* (Jurrus *et al.*, 2018 ▸) and displayed as ±2*k*_B_*T*/*e*. The portal region (lid and latch) and the entry portal are highlighted along with the latch residue Phe58 and the nonclassical NLS Lys21, Arg30 and Lys31. The portal region is positively charged (blue) with a ring of negative potential (red) below it.

In order to specify the regions with which hFABP4 binds to bicelles, a DMPG:DHCP *q*-value of 0.1 was chosen, as such mixed micelles are small enough to retain good resolution in the HSQC spectra. The resulting spectra were analyzed for the chemical shift changes as a function of sequence position and show that the lid and latch regions indeed exhibit the strongest chemical shift perturbations (Fig. 10 ▸), in accord with these regions attaching into one leaflet of the bicelle. Calculation of the electrostatic surface potential supports this membrane-binding mode. The calculated isoelectric point of hFABP4 is 7.2, but the charged residues are distributed unevenly on the surface. There are 20 Asp/Glu side chains, all of which are surface-exposed except Asp77, and 20 Arg/Lys side chains, of which 17 are surface-exposed (not Arg79, Arg107, and Arg127). The charges form a distinct pattern on the surface of hFABP4 with a positive patch at the portal region and a negatively charged belt below it (Fig. 10 ▸*d*). This asymmetric charge distribution may help in electrostatic steering of hFABP4 towards the membrane. Positive electrostatic potential clustered at the lid and latch regions has also been observed for FABP2 (LiCata & Bernlohr, 1998 ▸). In line with these notions and our NMR data, electrostatic potential calculations have shown that the association of FABPs with membranes is facilitated by nonspecific electrostatic inter­actions (Zamarreño *et al.*, 2012 ▸).

The mode of FABP4 binding to membranes has consequences for loading of hFABP4 with ligands. Two alternative mechanisms by which FABPs might retrieve a fatty acid have been proposed: (i) by diffusion of the fatty acid through the aqueous phase (Storch & Thumser, 2000 ▸) or (ii) by transient association of FABPs with membranes that contain dissolved fatty acids (Wootan *et al.*, 1993 ▸; Herr *et al.*, 1995 ▸; Corsico *et al.*, 1998 ▸; Liou & Storch, 2001 ▸). For fatty acids, the NMR data on hFABP4 would argue in favor of mechanism (ii), where a membrane-bound hFABP4 with the positively charged entrance region dipped into the negatively charged leaflet acquires a membrane-dissolved fatty acid (Fig. 10 ▸). In support of this notion, FABP7 can bind oleic acid and docosahexa­enoic acid micelles. Also, NMR data and multiscale molecular-dynamics simulations on FABP7 reveal that its interaction with micelles is through residues in the portal region (Lenz *et al.*, 2023 ▸). For inhibitors, the mechanism of FABP4 complexation would likely depend on the solubility of the small molecule. Hydrophobic inhibitors may dissolve in the membrane and be taken up by membrane-bound hFABP4, whereas soluble inhibitors may form complexes with hFABP4 in solution, possibly displacing a bound fatty acid.

#### 3.2.2. Ligand-dependent detachment of hFABP4 from bicelles and effect on the NLS

**Figure 11:**
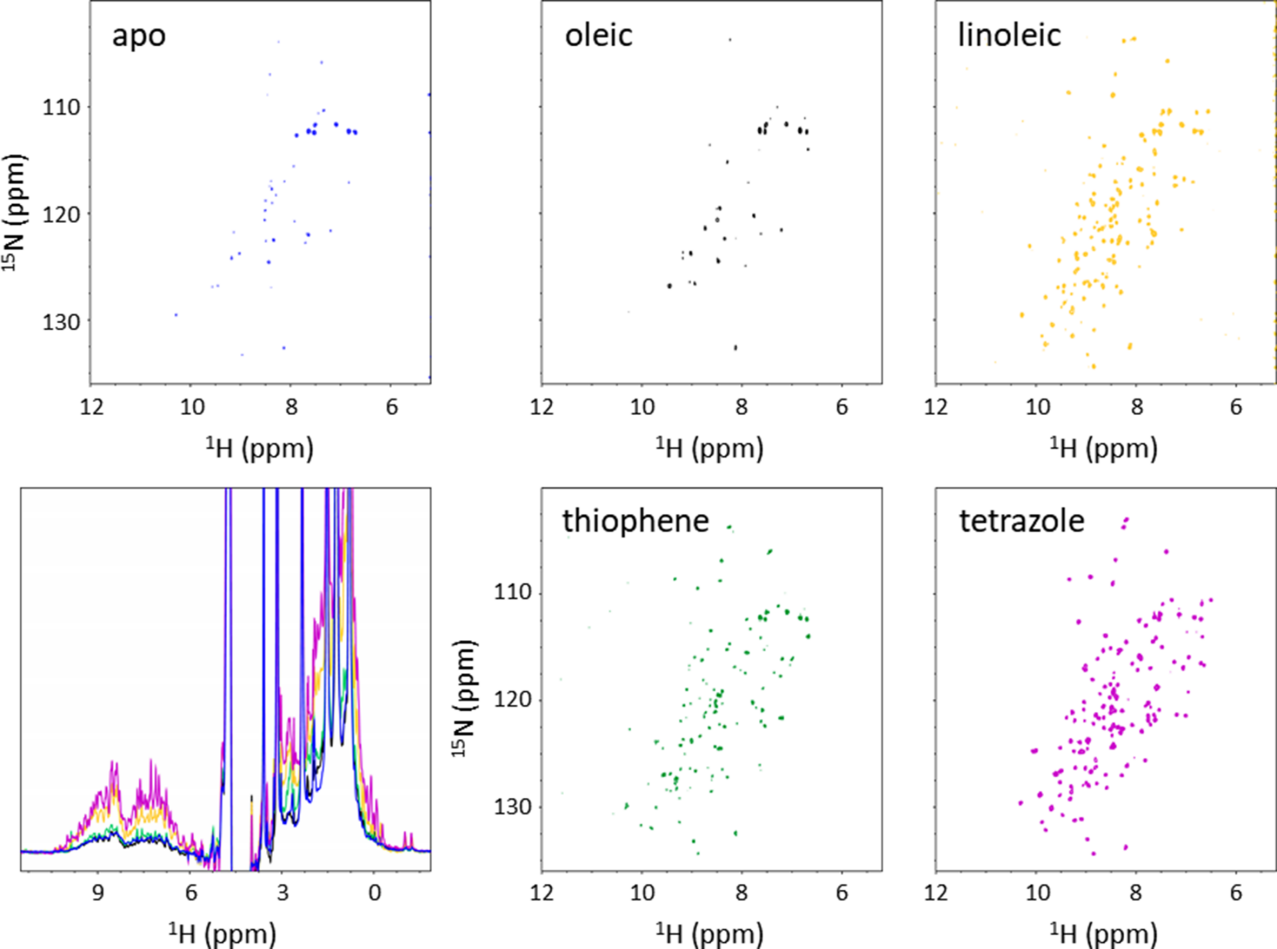
Response of hFABP4 to fatty-acid and inhibitor binding. The inhibitors are the same as in Fig. 8 ▸. The HSQC spectra are contoured at the same height for comparison. Apo hFABP4 (blue) strongly binds to bicelles (the same spectrum as in Fig. 9 ▸ for *q* = 0.75) and remains bound when oleic acid (black) is added. Upon the addition of a thiophene inhibitor (green), some detachment is apparent from the increased number and resolution of peaks. By contrast, the addition of linoleic acid (yellow) and a tetrazole inhibitor (magenta) lead to complete detachment of hFABP4 from micelles. The trend is also apparent from the 1D ^1^H NMR spectra, an enlarged version of which plus the corresponding orientations of Phe58 may be found in Supplementary Fig. S1. The line widths are smallest and the intensities are highest for linoleic acid and the tetrazole inhibitor, whereas for apo hFABP4 and oleic acid line broadening and bad peak resolution are strong. The thiophene inhibitor has intermediate properties.

Fatty acid-specific small changes in solvent accessibility have been observed for FABP2 (LiCata & Bernlohr, 1998 ▸). To test for similar effects in hFABP4, we tested the binding of hFABP4 to bicelles as a function of different ligands. Since delipidated hFABP4 was used in the studies, the effects observed by NMR can be attributed faithfully to the ligands added, excluding the effects of endogenously bound fatty acids. For these NMR measurements, charged DMPC:DHCP bicelles with *q* = 0.75 were chosen in order to maximize the differences in peak resolution between hFABP4 bound to bicelles and hFABP4 that is free in solution. Apo FABP4 strongly binds to bicelles, leading to the vanishing of most cross-peaks in the HSQC spectrum (Fig. 11 ▸; Supplementary Fig. S1). Upon the addition of either linoleic acid or a tetrazole inhibitor, sharp and well resolved cross-peaks are visible, indicative of detachment of hFABP4 from the bicelle with a concomitantly reduced tumbling time. By contrast, the individual addition of oleic acid or, to a lesser extent, a thiophene inhibitor do not change the appearance of the spectra, indicating that the hFABP4–ligand complex remains bound to the bicelle in these cases. Thus, it appears that the nature of the ligand modulates the affinity of hFABP4 for bicelles and hence potentially also for membranes.

Detachment of FABP4 from membranes is a prerequisite for nuclear import. It has been established that Phe58 in the latch region of FABP4 is an essential residue that adopts different conformations when FABP4 is in complex with different ligands (see also Fig. 7 ▸), and that ligand-stabilizing closure of Phe58 activates the protein for nuclear import, whereas the Phe58Ala variant does not exhibit nuclear import (Gillilan *et al.*, 2007 ▸). The nonclassical NLS of hFABP4 consists of the three positively charged residues Lys22, Arg31 and Lys32 clustered at the lid region that packs against the latch residue Phe58 when this side chain is in the *in*-conformation (Fig. 7 ▸). Thus, coupling of the conformation of Phe58 to the NLS in the lid region is a plausible molecular mechanism to activate hFABP4 for nuclear import. It has been suggested that voluminous ligands bound to FABP4 push against the portal region from the inside, inducing conformations that may lead to detachment from the membrane and/or nuclear import (Gillilan *et al.*, 2007 ▸). However, whereas the compact conformation of linoleic acid induces nuclear import, the extended conformation of oleic acid does not (Gillilan *et al.*, 2007 ▸). The compact conformation of linoleic acid with its two *cis* double bonds allows an *in*-conformation of Phe58, whereas oleic acid with a single *cis* double bond extends towards bulk solvent, pushing Phe58 into an *out*-conformation (Fig. 7 ▸*c*). It seems that the more exposed to the membrane Phe58 is, the higher the affinity of the hFABP4–ligand complex for membranes. This hypothesis is supported by the effect of hFABP4 inhibitors. The tetrazole locks Phe58 in the *in*-conformation with the phenyl side chain in van der Waals contact with the Cl atom of the inhibitor (Fig. 7 ▸*c*), triggering detachment of hFABP4 from bicelles. By contrast, the larger thiophene inhibitor results in a disordered latch region with no electron density for Phe58 (not shown in Fig. 7 ▸*c*) but allows hFABP4 to remain bound to the bicelle (Fig. 11 ▸). The position of Phe58 may be one indicator of the propensity of membrane detachment of hFABP4 but is likely not to be the only one. In the apo hFABP4 structure, which binds strongly to bicelles, Phe58 is in a very similar conformation as for the tetrazole inhibitor, which leads to detachment. Since crystal structures are limited in their prediction of flexibility, we speculate that in apo hFABP4 the absence of a ligand allows the latch to remain flexible when bound to membranes, such as to maximize membrane contact. The membrane-bound apo FABP4 would be uniquely suited as a resting state, waiting for the uptake of a membrane-dissolved ligand. Following ligand uptake, the nature of the ligand would then dictate the biological outcome by communication with the latch and the NLS. Future studies may include inhibitor-dependent nuclear import measurements as a proxy for gene expression, as these may prove to be very different across different chemical inhibitor series.

## 4. Conclusions and outlook

Starting from the observation that standard purified hFABP4 still contains a mixture of bound ligands, probably fatty acids, we developed a method to completely remove these ligands and thus prepared the protein for the study of the distinct effects that added ligands have on hFABP4. NMR studies, both in solution and with membrane-mimicking bicelles, showed a general rigidification of hFABP4 on ligand binding, especially at the lid and latch regions. These regions are also the contact sites of hFABP4 with micelles and bicelles, in accord with previous data. Binding of hFABP4 is strongest to negatively charged bicelles, which are accepted mimics of natural membranes. Membrane interaction is aided by the electropositive potential near the portal region of hFABP4, where the nonclassical NLS is also located. Such nonclassical three-dimensional NLS have been identified for human FABP isoforms 2, 4 and 5, and also for zebrafish FABP2 and the related human cellular retinoic acid-binding protein II (hCRAPBPII; Suárez *et al.*, 2020 ▸). Since all of the basic side chains comprising these NLS are clustered in the lid region, FABPs may well share a common mechanism for ligand-dependent membrane detachment and activation of nuclear import. Our NMR and crystallographic studies indicate that while the hydrophobic latch residue (Phe58 in hFABP4) is a major driver for coupling the nature of the bound ligand to the biological outcome, additional structural and dynamic parameters must be at play that are not yet fully understood.

## Supplementary Material

Excel file containing details of all crystal structures and IC50 values. DOI: 10.1107/S2059798325006242/gm5115sup1.xlsx

MTZ file for re-refined structure 2qm9. DOI: 10.1107/S2059798325006242/gm5115sup2.txt

Summary of quality criteria for 2qm9 and the re-refined model. DOI: 10.1107/S2059798325006242/gm5115sup3.txt

Supplementary Figure S1. DOI: 10.1107/S2059798325006242/gm5115sup4.pdf

## References

[bb1] Armstrong, E. H., Goswami, D., Griffin, P. R., Noy, N. & Ortlund, E. A. (2014). *J. Biol. Chem.* **289**, 14941–14954. 10.1074/jbc.M113.514646 PMC4031543 24692551

[bb2] Ayers, S. D., Nedrow, K. L., Gillilan, R. E. & Noy, N. (2007). *Biochemistry*, **46**, 6744–6752. 10.1021/bi700047a 17516629

[bb3] Bell, E., Ponthan, F., Whitworth, C., Westermann, F., Thomas, H. & Redfern, C. P. (2013). *PLoS One*, **8**, e68859. 10.1371/journal.pone.0068859 PMC3706415 23874790

[bb4] Bolterauer, C. & Heller, H. (1996). *Eur. Biophys. J.* **24**, 322–334.

[bb5] Cai, J., Lücke, C., Chen, Z., Qiao, Y., Klimtchuk, E. & Hamilton, J. A. (2012). *Biophys. J.* **102**, 2585–2594. 10.1016/j.bpj.2012.04.039 PMC3368126 22713574

[bb6] Caldwell, T. A., Baoukina, S., Brock, A. T., Oliver, R. C., Root, K. T., Krueger, J. K., Glover, K. J., Tieleman, D. P. & Columbus, L. (2018). *J. Phys. Chem. Lett.* **9**, 4469–4473. 10.1021/acs.jpclett.8b02079 PMC6353637 30024762

[bb7] Cavanagh, J., Fairbrother, W. J., Skelton, N. J. & Rance, M. (2018). *Protein NMR Spectroscopy: Principles and Practice*, 3rd ed. New York: Academic Press.

[bb8] Corsico, B., Cistola, D. P., Frieden, C. & Storch, J. (1998). *Proc. Natl Acad. Sci. USA*, **95**, 12174–12178. 10.1073/pnas.95.21.12174 PMC22804 9770459

[bb99] Ehler, A., Benz, J. & Rudolph, M. G. (2025). *Acta Cryst.* D**81**, 436–450. 10.1107/S2059798325005728 PMC12315584 40748030

[bb9] Furuhashi, M. & Hotamisligil, G. S. (2008). *Nat. Rev. Drug Discov.* **7**, 489–503. 10.1038/nrd2589 PMC2821027 18511927

[bb10] Gillilan, R. E., Ayers, S. D. & Noy, N. (2007). *J. Mol. Biol.* **372**, 1246–1260. 10.1016/j.jmb.2007.07.040 PMC2032018 17761196

[bb11] Glaser, S. T., Jayanetti, K., Oubraim, S., Hillowe, A., Frank, E., Jong, J., Wang, L., Wang, H., Ojima, I., Haj-Dahmane, S. & Kaczocha, M. (2023). *Sci. Rep.* **13**, 15234. 10.1038/s41598-023-42504-4 PMC10502087 37709856

[bb12] Herr, F. M., Matarese, V., Bernlohr, D. A. & Storch, J. (1995). *Biochemistry*, **34**, 11840–11845. 10.1021/bi00037a023 7547918

[bb13] Hotamisligil, G. S. & Bernlohr, D. A. (2015). *Nat. Rev. Endocrinol.* **11**, 592–605. 10.1038/nrendo.2015.122 PMC4578711 26260145

[bb14] Huang, P., Chandra, V. & Rastinejad, F. (2014). *Chem. Rev.* **114**, 233–254. 10.1021/cr400161b PMC3931982 24308533

[bb15] Ikura, M., Kay, L. E. & Bax, A. (1990). *Biochemistry*, **29**, 4659–4667. 10.1021/bi00471a022 2372549

[bb16] Johnson, B. A. & Blevins, R. A. (1994). *J. Biomol. NMR*, **4**, 603–614. 10.1007/BF00404272 22911360

[bb17] Jurrus, E., Engel, D., Star, K., Monson, K., Brandi, J., Felberg, L. E., Brookes, D. H., Wilson, L., Chen, J., Liles, K., Chun, M., Li, P., Gohara, D. W., Dolinsky, T., Konecny, R., Koes, D. R., Nielsen, J. E., Head–Gordon, T., Geng, W., Krasny, R., Wei, G. W., Holst, M. J., McCammon, J. A. & Baker, N. A. (2018). *Protein Sci.* **27**, 112–128. 10.1002/pro.3280 PMC5734301 28836357

[bb18] Kaczocha, M., Vivieca, S., Sun, J., Glaser, S. T. & Deutsch, D. G. (2012). *J. Biol. Chem.* **287**, 3415–3424. 10.1074/jbc.M111.304907 PMC3270995 22170058

[bb19] Kay, L. E., Ikura, M., Tschudin, R. & Bax, A. (2011). *J. Magn. Reson.* **213**, 423–441. 10.1016/j.jmr.2011.09.004 22152361

[bb20] Lenz, S., Bodnariuc, I., Renaud-Young, M., Butler, T. M. & MacCallum, J. L. (2023). *Biophys. J.* **122**, 603–615. 10.1016/j.bpj.2023.01.023 PMC9989940 36698315

[bb21] LiCata, V. J. & Bernlohr, D. A. (1998). *Proteins*, **33**, 577–589. 10.1002/(sici)1097-0134(19981201)33:4<577::aid-prot10>3.0.co;2-2 9849941

[bb22] Liou, H. L. & Storch, J. (2001). *Biochemistry*, **40**, 6475–6485. 10.1021/bi0101042 11371211

[bb23] Lücke, C., Rademacher, M., Zimmerman, A. W., Van Moerkerk, H. T. B., Veerkamp, J. H. & Rüterjans, H. (2001). *Biochem. J.* **354**, 259–266. 10.1042/0264-6021:3540259 PMC1221651 11171102

[bb24] Marr, E., Tardie, M., Carty, M., Brown Phillips, T., Wang, I.-K., Soeller, W., Qiu, X. & Karam, G. (2006). *Acta Cryst.* F**62**, 1058–1060. 10.1107/S1744309106038656 PMC2225221 17077479

[bb25] Mitchell, R. W., On, N. H., Del Bigio, M. R., Miller, D. W. & Hatch, G. M. (2011). *J. Neurochem.* **117**, 735–746. 10.1111/j.1471-4159.2011.07245.x 21395585

[bb26] Oliver, R. C., Lipfert, J., Fox, D. A., Lo, R. H., Kim, J. J., Doniach, S. & Columbus, L. (2014). *Langmuir*, **30**, 13353–13361. 10.1021/la503458n 25312254

[bb27] Piai, A., Fu, Q., Dev, J. & Chou, J. J. (2017). *Chemistry*, **23**, 1361–1367. 10.1002/chem.201604206 PMC5272838 27747952

[bb28] Rule, G. S. & Hitchens, T. K. (2006). *Fundamentals of Protein NMR Spectroscopy.* Dordrecht: Springer.

[bb29] Storch, J. & McDermott, L. (2009). *J. Lipid Res.* **50**, S126–S131. 10.1194/jlr.R800084-JLR200 PMC2674722 19017610

[bb30] Storch, J. & Thumser, A. E. (2000). *Biochim. Biophys. Acta*, **1486**, 28–44. 10.1016/s1388-1981(00)00046-9 10856711

[bb31] Suárez, M., Canclini, L. & Esteves, A. (2020). *PLoS One*, **15**, e0242312. 10.1371/journal.pone.0242312 PMC7660557 33180886

[bb32] Tan, N. S., Shaw, N. S., Vinckenbosch, N., Liu, P., Yasmin, R., Desvergne, B., Wahli, W. & Noy, N. (2002). *Mol. Cell. Biol.* **22**, 5114–5127. 10.1128/MCB.22.14.5114-5127.2002 PMC139777 12077340

[bb33] Wang, Q., Rizk, S., Bernard, C., Lai, M. P., Kam, D., Storch, J. & Stark, R. E. (2017). *Biochem. Biophys. Rep.* **10**, 318–324. 10.1016/j.bbrep.2017.05.001 PMC5614677 28955759

[bb34] Wootan, M. G., Bernlohr, D. A. & Storch, J. (1993). *Biochemistry*, **32**, 8622–8627. 10.1021/bi00084a033 8357805

[bb35] Zamarreño, F., Herrera, F. E., Córsico, B. & Costabel, M. D. (2012). *Biochim. Biophys. Acta*, **1818**, 1691–1697. 10.1016/j.bbamem.2012.03.003 22446190

