# Supplementary material for: A high-resolution data set of fatty acid-binding protein structures. I. Dynamics of FABP4 and ligand binding
Source: Acta Crystallogr D Struct Biol. 2025 Jul 28;81(Pt 8):423–35. doi: 10.1107/S2059798325006242 (PMC12315583; doi:10.1107/S2059798325006242)
Supplement: Supplementary file 4 [file d-81-00423-sup4.pdf]

STRUCTURAL
BIOLOGY

Volume 81 (2025)

Supporting information for article:

A high-resolution data set of fatty acid-binding protein structures. I. Dynamics of FABP4 and ligand binding

Fabio Casagrande, Andreas Ehler, Dominique Burger, Joerg Benz, Alfred Ross and Markus Rudolph

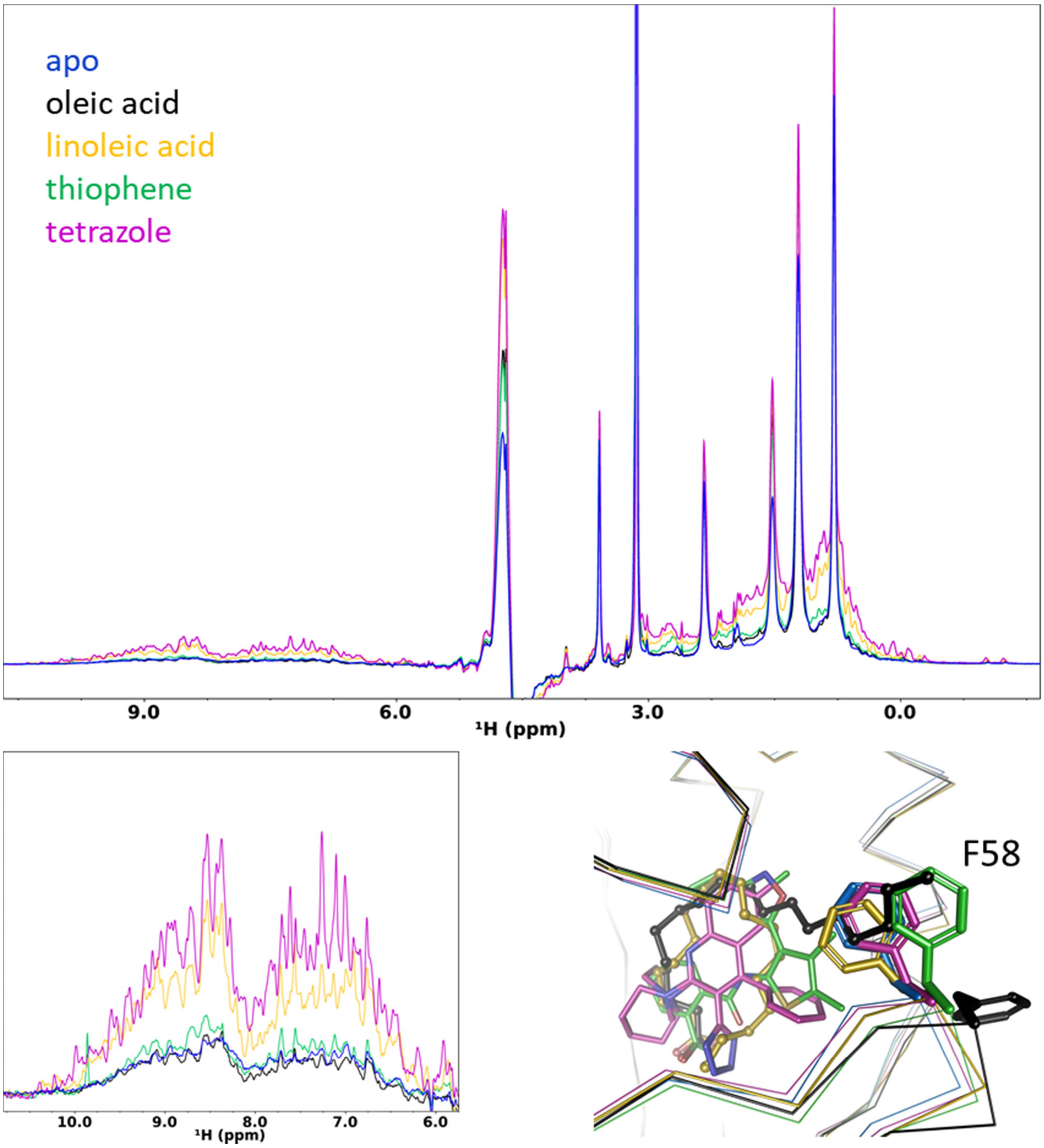

Figure S1 Enlarged 1D ^{1}H-NMR spectra and position of Phe58 in crystal structures. The spectrum of *in*- and *out*-conformations of this residue matches the peak width and resolution of the NMR spectra but for apo-FABP4 (blue), which adopts a conformation close to *in* but still binds to bicelles. Hence, elements in addition to Phe58 must determine the affinity of FABP4 to bicelles/membranes.
